# Examining Associations Between Child Abuse and Neglect Experiences With Emotion Regulation Difficulties Indicative of Adolescent Suicidal Ideation Risk

**DOI:** 10.3389/fpsyt.2021.630697

**Published:** 2021-04-06

**Authors:** Claire Hatkevich, Eric Sumlin, Carla Sharp

**Affiliations:** ^1^Department of Child and Adolescent Psychiatry and Behavioral Sciences, Children's Hospital of Philadelphia, Philadelphia, PA, United States; ^2^Department of Psychology, University of Houston, Houston, TX, United States

**Keywords:** emotion regulation, child trauma, abuse, neglect, adolescents, emotional abuse, suicide ideation

## Abstract

**Objective:** Preliminary work indicates one specific aspect of emotion dysregulation (i.e., limited access to emotion regulation strategies) uniquely associates with adolescent suicide ideation. An optimal score cut point on a measure of this emotion dysregulation impairment has been identified to indicate risk for past-year suicidal ideation. Examining types of child abuse and neglect associated with being above cut-off on this measure may point to interactive environmental effects associated with subsequent risk for suicidal ideation. The primary aim of this study was to investigate the relations between multiple types of child abuse and neglect with being above cutoff on a measure of limited access to emotion regulation strategies in a psychiatrically severe adolescent sample.

**Method:** The full sample included 203 psychiatric adolescents (Mean age = 15.31 years; 66.5% female; 74.4% White), assigned to two groups: (1) those at or above cutoff on the access to emotion regulation strategies subscale (*n* = 139); and (2) those below cutoff (*n* = 64).

**Results:** Significant differences were only evidenced between the emotion regulation cutoff groups on emotional abuse, after covarying for other types of abuse and neglect; significant group differences were not evidenced on any other type of abuse or neglect (sexual or physical abuse, emotional or physical neglect).

**Conclusion:** Relative to other types of abuse and neglect, emotional abuse may be differentially related to experiencing limited access to emotion regulation strategies, at the level indicative of suicide ideation risk. Clinical implications are discussed.

## Introduction

Emotion dysregulation, or “difficulties in emotion regulation,” is identified as a central, transdiagnostic risk factor for psychopathology in adolescence. Conceptualized as multifaceted difficulties in one's ability to identify, be aware of, accept, and modulate one's emotion appropriately and flexibly to a situation (1), emotion dysregulation has been indicated as an affective risk factor underlying many forms of psychopathology in adolescence [e.g., anxiety, eating pathology, aggressive behavior, post-traumatic stress disorder; (2, 3)]. One of the most concerning psychopathology outcomes that emotion dysregulation has been linked to is suicidal ideation, or thoughts about suicide (4). A substantial base of theoretical (5–7) and empirical research (8–10) implicates both broadband emotion regulation difficulties, as well as specific difficulties in emotion dysregulation, with increased risk for suicidal ideation, and this has been shown in adolescents specifically (11). Given the alarming prevalence of adolescent suicidal ideation [i.e., 18.8% considering a suicide attempt in the past-year; (12)], and rising rates of adolescents' hospitalization and emergency care visits for suicidal ideation (13), further investigation of affective risk factors (e.g., emotion dysregulation) for adolescent suicidal ideation, and especially factors which are malleable to intervention, is important.

One notable finding to emerge from this research indicates a unique association between suicidal ideation and one specific difficulty in emotion regulation, having limited access to emotion regulation strategies (i.e., referred to as “limited ERS” in the current paper). To illustrate, recent studies found that limited ERS, as captured with the Difficulties in Emotion Regulation Scale (DERS) “strategies” subscale (1), significantly predicted suicidal ideation, while covarying for all other emotion regulation difficulties and psychiatric diagnoses in both adolescents (11) and young adults (14). In other words, limited ERS differentially associates with suicidal ideation, when accounting for other types of emotion dysregulation (e.g., emotion non-acceptance, lack of clarity) and psychiatric disorder. Further, using receiver operating characteristic curve analysis, Hatkevich et al. (11) identified a score of 22.5 on a measure of limited ERS, the DERS strategies subscale, as the optimal cut-point for determining past-year suicidal ideation in a psychiatric adolescent sample. Taken together, research shows emotion dysregulation, and specifically having limited ERS at/or above the score of 22.5 on the DERS strategies subscale, confers risk for suicidal ideation.

Research is needed to better understand early-life risk factors associated with limited ERS and experiencing limited ERS at the level associated with suicidal ideation risk. Examining childhood-specific risk factors for limited ERS is vital, as emotional development and self-regulation in childhood provide the foundation upon which later adolescent emotion regulation is formed (15). Thus, examining emotion-relevant risk factors in childhood provides a key way that researchers can better understand the factors that place youth at-risk for limited ERS in adolescence, and better inform both prevention and intervention efforts.

One risk factor that has been extensively researched and linked to disruptions in emotional development and later emotion dysregulation is child maltreatment. Although definitions vary [see (15–17)], child maltreatment is generally conceptualized as the experience of any one of the following types of abuse and/or neglect in childhood (all definitions as proposed by 18, p. 175): (a) sexual abuse (i.e., sexual contact or conduct between a child and adult; 2003); (b) physical abuse (i.e., physical assault to a child with potential for injury; 2003); (c) physical neglect (i.e., caregiver failure to provide physical necessities, like housing or healthcare; 2003); (d) emotional abuse (i.e., any verbal acts that denigrate, humiliate, threaten, or otherwise devalue a child; 2003); or (e) emotional neglect (i.e., caregiver failure to provide emotional necessities, like love and support; 2003). Theory [e.g., (18, 19)] has long posited about the profound and deleterious impacts of child maltreatment on socioemotional, behavioral, and mental health development. In a developmental psychopathology model (15), child maltreatment is seen as a chronic “pathogenic environment” that impedes children in attaining fundamental developmental tasks (e.g., self-regulation, emotional development), and these disruptions carry forward and cascade through development, creating risk for later emotion dysregulation, maladaptive functioning, and psychopathology in adolescence (p. 414–415).

Consistent with theory, substantial research establishes child maltreatment as a salient risk factor for emotion dysregulation. Broadly, child abuse and neglect are linked with difficulties in emotion identification [e.g., alexithymia; (20)], lability (21), non-acceptance (22), and in use of adaptive emotion regulation strategies (23, 24). In line with developmental theory (15), this link has been demonstrated longitudinally, with early child maltreatment predicting greater emotion dysregulation in adolescence (25, 26). The robust nature of this relation was typified by a recent meta-analysis (27) finding that, across studies, maltreatment was significantly associated with both broadband emotion dysregulation, as well as specific difficulties in emotion regulation.

Emerging research in this area indicates that distinct child maltreatment experiences (i.e., different types of abuse and neglect) associate with different difficulties in emotion regulation. This was first demonstrated in longitudinal research by Egeland et al. (28), who found differences in developmental outcomes and emotion processes between different maltreatment groups (e.g., physical abuse; neglect).

Since this seminal work, trauma research has begun to further investigate how different types of child abuse/neglect associate with specific difficulties in emotion regulation. Although many forms of child trauma co-occur [i.e., “polyvictimization” “multi-type maltreatment”; (29, 30)], multiple findings have emerged about emotion regulation difficulties associated with specific forms of abuse/neglect. First, converging research indicates child physical abuse is linked to impairment in accurately identifying emotion/anger [e.g., inaccurately interpret hostile intent; (31)], and using more maladaptive emotion regulation strategies [e.g., aggressive and disruptive behavior; (31, 32). This is consistent with the experience of physical violence, and that emotion regulation develops in part through social learning and observing how parents/family members regulate emotion (33, 34).

Another notable finding to emerge is that emotional abuse and neglect appear to be associated with particularly severe and distinct patterns of emotion regulation deficits. Broadly, work (15, 35) links emotional neglect to greater impairment in emotion identification, including difficulty in identifying one's own emotions [i.e., alexithymia; (36)] and others' [e.g., reduced accuracy in identifying facial expressions and anger specifically; (37, 38)]. Further highlighting these emotion awareness deficits, Berzenski (35) found child emotion neglect was differentially associated with a factor comprised of difficulties in emotion clarity and awareness, but not with a factor comprised of other emotion regulation difficulties (e.g., in impulse control, goal-directed behavior, limited ERS). Making sense of these findings, Berzenski (35) and Cicchetti et al. (15) suggest emotion neglect, unlike abuse, presents an impoverished environment, where children lack the emotional “input” and modeling to learn to identify emotion [see (35)].

In contrast, emotional abuse has been more often linked to difficulties with using appropriate and adaptive emotion regulation strategies [i.e., “response-focused difficulties”; (39)]. Converging research (35, 40–42) suggests emotional abuse is more closely related to using maladaptive strategies to modulate affect, such as brooding/rumination (43), experiential avoidance (44), and with deficits engaging in goal-directed emotion-regulation behavior (45). In the first study to complexly account for the co-occurrence of other child trauma types in examining specific emotion regulation difficulties, Berzenski (35) found child emotional abuse was significantly associated with a factor represented by difficulties in limited ERS, using goal-directed behavior, and impulse control, but not with the factor (emotional awareness/clarity) linked to emotional neglect. Berzenski (35) and others (15, 41, 42) explain that emotional abuse environments likely provide youth with adequate opportunities to observe emotion, but are simultaneously characterized by modeling maladaptive emotion regulation strategies and reinforce youth in using maladaptive strategies themselves [e.g., emotion invalidation; (40)]. Taken together, emerging research indicates multiple forms of child abuse (physical, emotional) may be more closely related to deficits in implementing appropriate emotion regulation strategies/limited ERS, especially relative to emotional neglect, which appears to be more closely linked to deficits in emotion identification.

To our knowledge, no studies have bridged this research with newly emergent findings (11) that limited ERS uniquely associates with adolescent suicide ideation risk. Specific gaps exist in prior research and studies have not yet: (a) examined the differential relations of all child abuse/neglect types to limited ERS, accounting for other forms of child/abuse neglect; (b) investigated the association between different forms of child abuse/neglect and experiencing limited ERS above or below the level indicative of suicide ideation risk [22.5 DERS strategies score; (11)]; and (c) examined the aforementioned in a sample of clinically severe psychiatric adolescents with heterogeneous trauma presentations. Indeed, this is a critical priority addressed by prior studies (46). A comprehensive study which addresses these limitations has the potential to elucidate the unique associations of each child abuse/neglect type with limited ERS, and indicate which child abuse/neglect types may contribute to youth experiencing limited ERS at the level associated with adolescent suicide ideation risk. Empirical research identifying child abuse/neglect experiences associated with clear markers for suicide risk is important and has potential to inform early prevention and clinical intervention programs [e.g., in indicating the potential utility of trauma-focused interventions, like Trauma-Focused Cognitive Behavioral Therapy (TF-CBT) (47), to address affective suicide risk factors].

To this end, the current study will address these specific research gaps and study the differential relations between child abuse/neglect types and being above and below cutoff on a measure of limited ERS associated with suicide ideation risk, in a psychiatric adolescent sample. Specifically, the current study will investigate the differential relations of each trauma type to being above/below cutoff on limited ERS as captured with the DERS Strategies scale, while accounting for other trauma types concurrently. We will address these questions in a sample of psychiatric adolescents presenting to an inpatient treatment program. Based on prior aforementioned research, we expect that emotional and physical abuse will be significantly related to being above cutoff on limited ERS indicative of suicide ideation risk, while adjusting for other child trauma types. Given past work demonstrating emotional neglect is more closely related to emotion identification/awareness deficits [e.g., (35, 36)], we do not expect emotional neglect to be significantly related to being above/below cutoff on limited ERS, adjusting for other trauma types.

## Methods

### Participants and Procedures

#### Participants

Adolescents admitted to an inpatient treatment program serving youth with severe and treatment-refractory psychiatric disorders (i.e., those unremitted by prior treatments) were approached for consent. This treatment program specifically serves youth with a spectrum of co-occurring psychiatric diagnoses, though mood and anxiety-related disorders are amongst the most prevalent presenting diagnoses (see section Results for further details). Participants are a subset of participants who have completed the primary study measures from a larger study on assessment and treatment outcomes [see (48) for a description]. As the study battery was modified over time, there are subsets of adolescents who completed certain measures which were not administered to the full sample; 331 adolescents were administered the specific measures included in this study. Inclusion criteria for study participation were: (a) participants were aged between 12 and 17 years; (b) fluent in English; and (c) did not present with a psychotic-spectrum disorder or intellectual disability. Of the 331 adolescents approached for consent and participation, 58 were excluded from participation: 26 declined to participate, 15 did not meet eligibility criteria at admission, 8 were discharged before participation, 7 displayed signs of psychosis during assessment, 1 revoked consent, and 1 was excluded due to prior completion of a study battery including some of the same instruments. Of the remaining 273 adolescents, 203 fully completed the assessment battery and measures used in the current analyses, and were included in the final sample. The final sample consisted of *N* = 203 adolescents.

#### Procedures

This study was approved by the appropriate institutional review board. All assessments were conducted in private on the unit by trained clinical psychology doctoral students and clinical research assistants. Both parental consent and adolescent assent were obtained prior to assessment battery administration.

### Measures

#### Sociodemographic Information

Sociodemographic information (e.g., age, biological sex, race; household income) was obtained via a standard sociodemographic questionnaire and administrative intake.

#### Trauma History

Child abuse and neglect was assessed using the Childhood Trauma Questionnaire short form [CTQ-SF; (49)]. The CTQ-SF is a 28-item self-report based measure that asks participants to retrospectively recall how often they encountered or experienced various types of abuse and neglect; CTQ-SF items comprise five subscales of abuse and neglect, including: (a) sexual abuse (e.g., “Someone tried to touch me in a sexual way, or tried to make me touch them”); (b) physical abuse (e.g., “I got hit so hard by someone in my family that I had to see a doctor or go to the hospital”); (c) physical neglect (e.g., “I had to wear dirty clothes”); (d) emotional abuse (e.g., “People in my family said hurtful or insulting things to me”); and (e) emotional neglect (e.g., “My family was a source of strength and support,” reverse coded). Items are rated on a 5-point Likert scale (range: 1 “Never True,” to 5 “Very Often True”). Psychometric properties have been established for the CTQ-SF (49), and the CTQ-SF has been used with adolescent inpatients specifically [e.g., (50)]. Cronbach's alpha were as follows for CTQ-SF subscales in the current study: emotional abuse (α = 0.86), sexual abuse (α = 0.96), physical abuse (α = 0.63), emotional neglect (α = 0.90), and physical neglect (α = 0.54).

#### Difficulties in Emotion Regulation

Difficulties in emotion regulation, including limited ERS specifically, were measured with the Difficulties in Emotion Regulation Scale [DERS; (1)]. The DERS is a 36-item self-report based measure that assesses six facets of emotion dysregulation, consistent with the multidimensional conceptualization proposed by Gratz and Roemer (1): (a) lack of emotional awareness (awareness); (b) lack of emotional clarity (clarity); (c) non-acceptance of emotional states (non-acceptance); (d) difficulties engaging in goal-directed behaviors (goals); (e) impulse control difficulties (impulse); and (f) limited ERS (DERS “strategies” subscale). Participants respond to all items on a 5-point Likert scale, and indicate how much each item pertains to them (1 = “almost never,” to 5 = “almost always”), and items are summed onto each of the six aforementioned subscales; higher scores on DERS subscales indicate greater emotion regulation impairment. For the current study, all subscales were included for descriptive purposes, though the strategies subscale capturing limited ERS was the focus of primary analyses. As aforementioned, prior work (11) conducted receiving operating characteristics and identified a score of 22.5 on the DERS strategies scale as the optimal cut-point (i.e., having maximal sensitivity and specificity) for determining past-year suicidal ideation; for the cut point of 22.5, sensitivity was 74.3% and specificity was 64.1%. The cut-point of 22.5 on DERS strategies was used to divide the sample into the following groups: (1) those at or above cutoff for limited ERS, indicative of risk for suicidal ideation; and (2) those below cutoff. Psychometric properties of the DERS are well-established in adolescent populations [e.g., (10, 51)], with its factor structure validated in adolescent inpatients specifically (52). Reliability was α = 0.905 for the DERS strategies subscale in the current study.

#### Psychiatric Diagnoses

Psychiatric diagnoses were assessed with the Computerized Diagnostic Interview Schedule for Children [CDISC; (53)], a structured diagnostic interview with well-established psychometric properties (53, 54), which has been used in a number of psychiatric adolescent samples [e.g., (55, 56)]. The CDISC assesses for the presence of psychiatric diagnoses consistent with DSM-IV, and is administrated by a trained clinician and/or assessor. In the current study, presence of any Mood Disorder, Externalizing Disorder, and Anxiety Disorder diagnoses were included in descriptive, bivariate and *post-hoc* analyses, given the high rates of psychiatric disorder and comorbidity of youth presenting to this psychiatric care setting. Any Mood Disorder diagnosis was defined as meeting criteria for Major Depressive Disorder, Dysthymia, Hypomania, and/or Mania. Any Anxiety Disorder diagnosis was defined as meeting criteria for Generalized Anxiety Disorder, Specific Phobia, Social Phobia, Separation Anxiety Disorder, Obsessive-Compulsive Disorder, Post-traumatic Stress Disorder, Panic Disorder, and/or Agoraphobia. Any Externalizing Disorder was defined as meeting criteria for Oppositional Defiant Disorder, Conduct Disorder, and/or Attention-Deficit/Hyperactivity Disorder. Psychometric properties have been well-established for this measure, with interrater reliability calculated between 0.91 and 1.00 for all disorders (57), and excellent test-retest reliability (58).

#### Data Analytic Strategy

All analyses were conducted in SPSS statistical software. Descriptive statistics were used to examine characteristics of main study variables (CTQ-SF abuse/neglect scales, DERS subscales), sociodemographics (age, sex, race, household income), and clinical characteristics (psychiatric diagnoses) of the full sample. As described in the methods, participants in the full sample were classified into two groups based on their score on the DERS strategies subscale: (1) Participants at or above cutoff for limited ERS, indicative of risk for suicidal ideation (referred to as the limited ERS above cutoff group); and (2) Participants below cutoff (referred to as the limited ERS below cutoff group). These two groups were then used in subsequent and primary analyses. Bivariate analyses (i.e., bivariate correlations, independent sample *t*-tests, chi-square analyses) were conducted to examine relations amongst main study variables, sociodemographic, and clinical characteristics. For our primary analyses, we conducted a series of analyses of covariance (ANCOVA) to examine group differences between the limited ERS groups on each CTQ-SF abuse/neglect scales (emotional abuse, emotional neglect, physical abuse, physical neglect, sexual abuse), while covarying for each of the remaining CTQ-SF abuse/neglect scales. In each respective model with CTQ-SF abuse/neglect scale as the dependent variable, we included the other four remaining CTQ-SF abuse/neglect scales as covariates (see below for rationale). Given sample size and power considerations, we originally did not include additional clinical or sociodemographic variables as covariates in our primary ANCOVA models, with exception of the model examining CTQ-SF sexual abuse as the outcome, in which we included biological sex as an additional covariate to the remaining CTQ-SF abuse/neglect scales. For ANCOVA models, adjusted means with Bonferroni corrections, standard error, and pairwise comparisons were calculated.

## Results

### Full Sample Demographic and Clinical Characteristics

The full sample consisted of 203 adolescent inpatients completing all primary study measures and meeting inclusion criteria. Demographic and clinical characteristics are fully depicted in Table 1. Demographically, the full sample was 66.5% female, 74.4% White/Caucasian, with a mean age of 15.31 years, and the majority of participants' household incomes were above $100,000. Clinically, as determined on the CDISC interview, 70.9% of the sample met diagnostic criteria for any Mood Disorder diagnosis, 69% met for any Anxiety Disorder diagnosis, and 42.9% met for any Externalizing Disorder diagnosis.

**Table 1:** Full sample demographic and clinical characteristics.

	**Full sample (*n* = 203)**
Patient age	15.31 (1.43)
**Race**	
White/Caucasian	151 (74.4%)
American Indian or Alaskan Native	1 (.5%)
Asian	6 (3%)
Black or African American	2 (1%)
Multiracial or other-identified	13 (6.4%)
Missing data	30 (14.8%)
**Biological sex**	
Female	135 (66.5%)
Male	68 (33.5%)
**CDISC psychiatric diagnoses**	
Any mood disorder	144 (70.9%)
Any anxiety disorder	140 (69%)
Any externalizing disorder	87 (42.9%)
**Household income**	
Below $100,000	11.5%
Above $100,000	66.3%
Missing data/decline to answer	22.2%

*Age data is depicted in years, with the mean age followed by standard deviation in parentheses. Race and Biological Sex data depicted is the number of youth reporting each identity, followed by the percent of the full sample with this identity depicted in parentheses. CDISC Psychiatric Diagnoses data depicts first the number of youth meeting criteria for each diagnosis, followed by the percent of the full sample meeting criteria for the diagnosis. Household income data depicts percentage of the full sample identifying in the following income brackets, and lastly those with missing income data or declining to answer*.

### Descriptive Statistics for Main Study Variables

Means, standard deviations and ranges for CTQ-SF abuse/neglect scales and DERS subscales are depicted in Table 2. For the CTQ-SF scales, highest mean scores were reported for emotional neglect (*M* = 11.11; SD = 5.04) and emotional abuse (*M* = 10.26; SD = 5.23), with all other scale means between 6.49 (physical abuse) and 7.16 (physical neglect). For the full sample, 76.8% of the sample endorsed some experience of emotional abuse, 83.7% of some emotional neglect, 42.4% of some physical abuse, 66% of some physical neglect, and 16.7% of some experience of sexual abuse; these percentages specifically depict the portion of participants who endorsed any non-zero response on each of the aforementioned CTQ-SF scales. For the DERS subscales, the highest overall mean was indicated for the primary variable of interest, limited ERS (Strategies subscale *M* = 26.62), followed by lack of emotional awareness, difficulties engaging in goal-directed behavior, and non-acceptance of emotional responses.

**Table 2:** Descriptive statistics for CTQ-SF abuse and neglect scales and DERS subscales.

**Study variable**	**Mean**	**Standard deviation**	**Range**
CTQ-SF emotional abuse	10.26	5.23	5–25
CTQ-SF physical abuse	6.49	2.58	5–17
CTQ-SF sexual abuse	6.55	4.61	5–25
CTQ-SF emotional neglect	11.11	5.04	5–24
CTQ-SF physical neglect	7.16	2.59	5–21
DERS non-acceptance	17.15	7.18	6–30
DERS goals	18.97	5.09	5–25
DERS impulse	16.51	6.65	6–30
DERS awareness	18.97	5.96	6–30
DERS strategies	26.62	8.30	8–40
DERS clarity	15.89	5.19	5–25

*CTQ-SF, Childhood Trauma Questionnaire short form; DERS, Difficulties in Emotion Regulation Scale, each DERS subscale is listed above and is described in the Methods section*.

As described in the section Methods, participants in the full sample (*N* = 203) were assigned to the following groups, based on their DERS strategies score: (1) limited ERS above cutoff group (*n* = 139; 68.5% of full sample); and (2) limited ERS below cutoff group (*n* = 64; 31.5% of full sample).

### Bivariate Relations

Bivariate correlations were conducted to examine associations between all CTQ-SF abuse/neglect scales, DERS strategies, age in years, and any CDISC mood, anxiety, and externalizing diagnoses. Correlational analyses are depicted in Table 3. In summary, age was not significantly correlated with any CTQ-SF abuse/neglect scale, nor with DERS strategies or any CDISC diagnoses. DERS strategies was significantly positively correlated with CTQ-SF scales of emotional abuse (*r* = 0.28; *p* < 0.001), emotional neglect (*r* = 0.23; *p* = 0.001), and physical neglect (*r* = 0.15; *p* = 0.032), and with CDISC mood and anxiety diagnoses, such that greater limited ERS was associated with these diagnoses and forms of child abuse/neglect. With regards to CDISC diagnoses, mood and anxiety diagnoses shared no significant relations with any CTQ-SF abuse/neglect subscale; in contrast, CDISC externalizing diagnosis was significantly related to all forms of CTQ-SF abuse/neglect, except sexual abuse. In examining correlations between the CTQ-SF abuse/neglect scales, significant positive correlations exist between all CTQ-SF abuse and neglect scales (physical abuse, physical neglect, emotional abuse, emotional neglect, sexual abuse), with effect sizes ranging between 0.22 and 0.71, with most values between 0.2 and 0.5 (see Table 3).

**Table 3:** Bivariate correlation matrix.

		**1**	**2**	**3**	**4**	**5**	**6**	**7**	**8**	**9**	**10**
1	Emotional abuse	-									
2	Physical abuse	0.51^**^	-								
3	Sexual abuse	0.40^**^	0.41^**^	-							
4	Emotional neglect	0.71^**^	0.34^**^	0.22^**^	-						
5	Physical neglect	0.45^**^	0.32^**^	0.26^**^	0.55^**^	-					
6	DERS strategies	0.28^**^	−0.01	0.06	0.23^**^	0.15^*^	-				
7	Age	0.02	0.01	−0.00	−0.04	−0.07	−0.10	-			
8	Mood Dx.	0.06	−0.02	0.06	0.05	−0.03	0.42^**^	0.04	-		
9	External. Dx.	0.26^**^	0.19^**^	−0.01	0.19^**^	0.18^**^	0.09	0.05	−0.02	-	
10	Anxiety Dx.	0.10	0.06	0.12	0.02	0.03	0.20^**^	0.05	0.25^**^	0.09	-

*Data are bivariate Pearson correlations. 1–5 are from the CTQ-SF measure. 8–10 are from the CDISC interview, with Mood Dx., CDISC any mood diagnosis; External. Dx., CDISC any externalizing diagnosis; and Anxiety Dx., CDISC any anxiety diagnosis*.

** *Correlation is significant at the 0.01 level (2-tailed)*.

* *Correlation is significant at the 0.05 level (2-tailed)*.

Further bivariate analyses were conducted to examine the relations between biological sex, limited ERS cutoff groups, and CTQ-SF abuse/neglect scales. Chi square analyses indicated there was no significant association between biological sex and limited ERS cutoff group assignment, χ^2^(1) = 0.672, *p* = 0.412. Independent sample *t*-tests were conducted to examine the relation between biological sex and CTQ-SF abuse/neglect scales. Results indicated no significant sex differences existed for all CTQ-SF abuse/neglect subscales, with exception of sexual abuse [*t*(191.570) = 3.388, *p* =.001], such that females reported significantly greater sexual abuse experiences (*M* = 7.15, SD = 5.362), than males (*M* = 5.37, SD = 2.073). Due to extremely small cell sizes for groups identifying racial identities other than White/Caucasian racial identity (e.g., 1 individual identitying as American Indian/Alaskan Native, 6 identifying as Asian), we were not sufficiently powered to conduct group analyses examining the link between racial identity and main study variables (e.g., CTQ-SF abuse/neglect scales, limited ERS cutoff groups).

Overall, bivariate analyses indicated that CTQ-SF abuse and neglect scales (i.e., physical abuse, physical neglect, emotional abuse, emotional neglect, sexual abuse) are significantly correlated with one another, consistent with research indicating individuals experiencing child abuse and neglect are likely to experience multiple forms of abuse and neglect, or “multi-type maltreatment” [e.g., (30)]. In order to account for this overlap in subsequent analyses, and thus examine the unique relation of each abuse/neglect type to limited ERS cutoff group in sequence, remaining types of abuse/neglect were included as covariates in our primary analyses; this is consistent with the analytic approach of other child maltreatment research [e.g., (59)]. In regards to sociodemographics, age was not significantly related to limited ERS as captured by DERS strategies, or any CTQ-SF abuse/neglect type, and biological sex was not significantly associated with limited ERS cutoff group, nor any form of abuse/neglect, with exception of sexual abuse. As a result of sex differences evident for sexual abuse, we additionally elected to include biological sex as a covariate in our ANCOVA with sexual abuse as outcome.

Importantly, in bivariate relations, CDISC psychiatric diagnoses shared multiple significant relations with main study variables (e.g., any mood and anxiety diagnosis were significantly related to limited ERS, and any externalizing diagnosis was significantly related to most types of child abuse and neglect). Due to sample size and power considerations, we elected to follow our original analytic plan, and first test the aforementioned ANCOVA models examining limited ERS group differences for each CTQ-SF abuse/neglect type, without any additional psychiatric diagnosis covariates. In order to further consider potential links between CDISC psychiatric diagnoses, limited ERS group status, and CTQ-SF abuse/neglect, we then conducted additional exploratory *post-hoc* analyses (see below for further detail).

### Primary Analyses—ANCOVA Models

We conducted a series of five analysis of covariance (ANCOVA) models to examine group differences between the groups above and below cutoff on limited ERS, on each respective CTQ-SF abuse and neglect scale. In each model, we included the other CTQ-SF abuse/neglect scales as covariates, and additionally included biological sex as a covariate in the ANCOVA model with sexual abuse as outcome.

Results of the ANCOVA models are depicted in Table 4. In the model examining CTQ-SF emotional abuse as outcome, a significant difference was evidenced between groups [*F*_(1, 197)_ = 4.452, *p* = 0.036, partial η^2^ = 0.022], after covarying for other CTQ-SF abuse/neglect scales, such that the limited ERS above cutoff group reported significantly greater emotional abuse (adjusted *M* = 10.599), than the limited ERS below cutoff group (adjusted *M* = 9.528). In the model examining CTQ-SF emotional neglect as outcome, no significant differences were evidenced between limited ERS above and below cutoff groups on emotional neglect [*F*_(1, 197)_ = 0.426, *p* = 0.515, partial η^2^ = 0.002], after adjusting for other CTQ-SF abuse/neglect scale covariates. In the model examining CTQ-SF physical abuse as outcome, no significant differences were evidenced between limited ERS above and below cutoff groups on physical abuse [*F*_(1,197)_ = 2.223, *p* = 0.138, partial η^2^ = 0.011], after adjusting for other CTQ-SF abuse/neglect scale covariates. In the model examining CTQ-SF physical neglect as outcome, no significant differences were evidenced between limited ERS above and below cutoff groups on physical neglect [*F*_(1,197)_ = 0.000, *p* = 0.991, partial η^2^ = 0.000], after adjusting for other CTQ-SF abuse/neglect scale covariates. Lastly, in the model examining CTQ-SF sexual abuse as outcome, no significant differences were evidenced between limited ERS above and below cutoff groups on sexual abuse [*F*_(1,196)_ = 0.273, *p* = 0.602, partial η^2^ = 0.001], after adjusting for other CTQ-SF abuse/neglect scales and biological sex. In summary, significant differences were only evidenced between the limited ERS above vs. below cutoff groups on the CTQ-SF trauma scale of emotional abuse, after covarying for other types of CTQ-SF abuse and neglect; significant group differences were not evidenced on any other form of CTQ-SF trauma [emotional neglect, physical abuse, physical neglect, sexual abuse], after adjusting for all other types of abuse/neglect.

**Table 4:** ANCOVA results showing CTQ-SF abuse/neglect scale outcomes for the limited ERS above and below cutoff groups.

**CTQ-SF abuse/neglect scale**	**Limited ERS above cutoff group (*n* = 139)**	**Limited ERS below cutoff group(*n* = 64)**	***F***	***p***	**Partial eta square*d* (η^2^)**
	***M* (SD)**	***M* (SD)**			
Emotional abuse	10.599 (0.280)	9.528 (0.417)	4.452	0.036	0.022
Emotional neglect	11.215 (0.283)	10.878 (0.423)	0.426	0.515	0.002
Physical abuse	6.331 (0.183)	6.828 (0.273)	2.223	0.138	0.011
Physical neglect	7.159 (0.185)	7.155 (0.276)	0.000	0.991	0.000
Sexual abuse	6.655 (0.344)	6.327 (0.514)	0.273	0.602	0.001

*N = 203 youth were included in ANCOVAs. All ANCOVAs included all other CTQ-SF Abuse/Neglect scales as covariates (see section Methods for further description), and the ANCOVA with CTQ-SF Sexual Abuse additionally included sex as a covariate. Data shown for the CTQ-SF Abuse/Neglect scales are adjusted means. CTQ-SF, Childhood Trauma Questionnaire short form*.

Across all ANCOVA models, other CTQ-SF abuse/neglect scale covariates emerged as significant predictors of the examined CTQ-SF abuse/neglect outcome (e.g., in the emotional neglect outcome model, the covariates of emotional abuse and physical neglect were significant predictors, with greater emotional abuse and physical neglect significantly predicting greater emotional neglect).

### Post-hoc Analyses

Additional exploratory *post-hoc* analyses were conducted based on our bivariate and primary ANCOVA model findings. Given that bivariate analyses revealed multiple significant relations between CDISC psychiatric diagnoses (mood, anxiety, externalizing) and primary study variables (limited ERS, CTQ-SF abuse/neglect scales), we elected to conduct additional analyses to examine CDISC psychiatric diagnoses as additional covariates in models examining the link between limited ERS group status and CTQ-SF abuse/neglect. Given our primary ANCOVA results that significant group differences were only evidenced for the CTQ-SF emotional abuse scale, but not other types of abuse/neglect, we conducted two additional *post-hoc* ANCOVA models examining limited ERS group differences on emotional abuse specifically: (a) in the first, we included all original covariates (i.e., other CTQ-SF abuse/neglect types: emotional neglect, physical abuse, physical neglect, sexual abuse) and CDISC any mood diagnosis as an additional covariate; and (b) in the second model, we included all the aforementioned CTQ-SF abuse/neglect scales, and three psychiatric diagnoses (CDISC any mood, anxiety, and externalizing) as covariates.

In the first *post-hoc* ANCOVA model with CDISC any mood diagnosis and all other CTQ-SF abuse/neglect scales (physical abuse, physical neglect, emotional neglect, sexual abuse) as covariates, significant group differences were found for limited ERS status groups on emotional abuse [*F*_(1,196)_ = 4.125, η^2^ = 0.021, *p* = 0.044], even while covarying for any mood disorder diagnosis and all other types of CTQ-SF abuse/neglect. Notably, the effect size of this significant finding remained almost the same as compared to the original ANCOVA model without CDISC any mood diagnosis included as a covariate.

In the second *post-hoc* ANCOVA model with the covariates of three psychiatric diagnoses (CDISC any mood, anxiety, and externalizing diagnoses) and all remaining CTQ-SF abuse/neglect scales (physical abuse, physical neglect, emotional neglect, sexual abuse), no significant differences were evidenced between limited ERS groups on emotional abuse [*F*_(1,194)_ = 2.831, η^2^ = 0.014, *p* = 0.094]. Of the CDISC psychiatric diagnoses included as covariates, only CDISC any externalizing disorder diagnosis was significant [*F*_(1,194)_ = 4.906, η^2^ = 0.025, *p* = 0.044] in predicting the outcome, CTQ-SF emotional abuse. Observed power was calculated, given our addition of three covariates to the original model and initial power concerns. Importantly, the observed power for examining limited ERS group differences on emotional abuse was 0.388, which is below the accepted value of 0.80 for adequate power. Thus, there was inadequate power to detect a true effect of limited ERS group differences on emotional abuse, in this model with three additional psychiatric diagnosis covariates.

## Discussion

The current study examined the differential relations between child abuse/neglect types and being above and below cutoff on a measure of limited ERS associated with suicide ideation risk, in a psychiatric adolescent sample. For primary analyses, we conducted a series of ANCOVAs to examine differences between the limited ERS groups (above, below cutoff) on each CTQ-SF abuse/neglect scales (emotional abuse, emotional neglect, physical abuse, physical neglect, sexual abuse), while covarying for all other CTQ-SF abuse/neglect scales. Results revealed significant differences for limited ERS groups on only the CTQ-SF emotional abuse scale, when covarying for other types of abuse and neglect, and significant group differences were not evidenced for any other type of abuse or neglect (sexual or physical abuse, emotional or physical neglect). Addressing critical gaps in prior research, this study represents a novel first-look at the differential associations of various forms of child abuse and neglect to a clear affective risk marker (i.e., a cut-point on limited ERS) for adolescent suicide ideation; further, it extends prior work conducted in undergraduate samples (35, 41) to a high-risk sample of psychiatric youth with diverse clinical and trauma presentations.

Our primary finding that emotional abuse is the only trauma type significantly related to being above cutoff on limited ERS converges with prior theoretical and empirical research. This finding, which persisted even after accounting for other types of child abuse and neglect, closely mirrors results of one key study informing this work, Berzenski (35). Akin to our findings, Berzenski (35) showed that emotional abuse was most closely linked to an emotion regulation difficulties factor comprised of limited ERS and other behavior-regulation difficulty subscales of the DERS; importantly, other trauma types (i.e., emotional neglect) were not related to this overarching factor. Our pattern of results parallel these findings, and indicate emotion abuse is differentially related to limited ERS, relative to other types of abuse and neglect.

The fact that emotional abuse was uniquely linked to experiencing limited ERS at the level indicative of suicide risk falls in line with literature documenting the severe and deleterious impacts of emotional abuse on both mental health broadly and emotion regulation specifically. Although all forms of child maltreatment have adverse impacts on child development (60), emerging studies (59, 61–63) show that emotional abuse has especially deleterious and long-lasting effects on mental health, emotion functioning, and suicide outcomes. Substantial literature (15, 35, 40–42)] documents the profound impact emotion abuse has on emotion regulation specifically. Prior work establishes that emotional abuse impairs emotion regulation in a multitude of ways, through its chronicity (64), ruptures and betrayal in the primary attachment relationship (45, 65), and early disruptions in emotion regulation and the development of internal working models (15, 42), which crystallize through development and form the basis for later emotion dysregulation (15). In whole, our finding that emotional abuse uniquely associates with limited ERS at the level associated with suicide ideation risk converges with this large body of work demonstrating the profound impacts of emotional abuse on developing emotion regulation. It also converges with specific work (34, 35, 66) suggesting that environments characterized by emotional abuse likely include modeling of maladaptive emotion regulation strategies, punishment/invalidation of appropriate emotional expression, which subsequently leave youth with limited ERS.

In contrast, all other forms of abuse/neglect (i.e., emotional neglect, physical abuse, physical neglect, and sexual abuse) did not significantly differ by limited ERS cutoff groups, when accounting for other trauma types in each respective model. An interesting and complicated pattern of findings emerged for emotional neglect specifically: at the bivariate level, emotional neglect was significantly associated with limited ERS, such that greater emotional neglect was related to more impairment in limited ERS; however, when covarying for other trauma types, ANCOVA results indicated no significant limited ERS group differences by emotional neglect. Taken together, this pair of findings may suggest that emotional neglect *is* linked to limited ERS, but it does not appear to be associated with limited ERS at the level indicative of suicide ideation risk, or when other trauma types are accounted for. Given work [e.g., (35, 36, 38)] suggesting emotion neglect is more closely linked to emotional identification difficulties, it is surprising and counter to our initial hypotheses that emotional neglect related with limited ERS in correlational results. One potential explanation is that emotionally-absent caregiving experiences may also leave youth to develop their own emotion regulation strategies, without instruction in how to “match” appropriate strategies to context, or with strategies that are maladaptive/socially-unacceptable, but insufficient caregiver correction is provided over time.

No significant differences were evidenced for limited ERS cutoff groups on physical abuse, physical neglect, or sexual abuse, when other trauma types were accounted for. One tentative explanation for these findings is that perhaps these forms of trauma are not unrelated to limited ERS, but that these relations may be only be evident when studied in specific diagnostic subsamples. To illustrate, Jennissen et al. (67) found significant relations between all CTQ-SF subscales and emotion regulation difficulties on the DERS in adults with diverse psychopathology, whereas a more nuanced pattern of relations between forms of abuse/neglect and emotion regulation difficulties emerged in samples with particular diagnoses. For example, in adults with substance use disorders, physical abuse was significantly related to emotion regulation difficulties in goal-directed behavior and impulse control, and sexual abuse to no emotion regulation difficulties (68). Taken together, these results may suggest that for certain forms of child trauma, like physical and sexual abuse, relations with emotion regulation deficits may be most evident in diagnostic-specific subsamples; for example, the relations of sexual abuse and emotion regulation deficits may differ when examined in youth with trauma and stressor-related disorders specifically. Alternatively, it may also be the case that physical abuse, physical neglect, and sexual abuse are better typified by other patterns of adolescent emotion regulation deficits, and not limited ERS, as examined in the current study. Elucidating these questions and the nature of our findings will be a key next step for future research.

In a highly preliminary attempt to address the potential role of psychiatric diagnoses in our primary study findings, exploratory *post-hoc* analyses were conducted to sequentially account for various psychiatric diagnoses (mood, anxiety, externalizing) as covariates in ANCOVA models examining limited ERS group differences on emotional abuse. *Post-hoc* analyses indicated that group differences remained when mood disorder alone was included alongside CTQ-SF abuse/neglect types, but not in the model including all forms of psychiatric diagnoses concurrently (i.e., mood, anxiety, and externalizing diagnoses together). Unfortunately, due to being significantly under-powered to detect a true effect in our latter model, our study is ultimately unable to definitively speak to whether significant limited ERS group differences persist for emotional abuse, when covarying for mood, anxiety, and externalizing psychopathology concurrently. Given the potential role of psychopathology in the relation between limited ERS and child trauma experiences, this will be a key direction for future research in a larger psychiatric adolescent sample.

### Limitations

The current study has multiple limitations and related implications for future research. First, our study elected to investigate the links between specific child trauma types and a limited ERS risk indicator for suicide ideation, but did not examine suicide ideation specifically. This study was designed to primarily investigate the potential early child maltreatment experiences associated with this affective risk factor, while accounting for other forms of child trauma. Thus, while suicide ideation is relevant as the ultimate risk outcome for this emotion regulation impairment, examining ideation directly moved beyond the scope of the current study. A critical next step will be to longitudinally examine the links between childhood emotional abuse, adolescent limited ERS, and either concurrent or subsequent suicidal ideation. Second, demographically, our study was predominantly white (74.4%), which limits the generalizability of our findings. Prior research (69) highlights the differences in emotion socialization for European American, African American, Asian American, and Latin American families, and indicates that emotion-related parenting practices may differentially impact outcomes in youth of varying backgrounds. Thus, it will be important for future research to replicate these findings in an ethnoracially diverse youth sample. Lastly, adolescents were recruited from a psychiatric treatment unit designed to address treatment-refractory mental illness; thus, although this sample is psychiatrically complex, the majority of participants have previously received treatment, and likely differ from youth with equally complex clinical and trauma histories who have not previously obtained mental health services.

### Clinical Implications

Notwithstanding these limitations, the current study has important strengths and implications for prevention and intervention efforts. Notably, the current study is the first known investigation to investigate which child abuse/neglect types associate with youth experiencing limited ERS at the level conferring risk for suicide ideation. Our study identified preliminary evidence that emotional abuse may be a unique child trauma associated with specific emotion regulation impairments indicative of suicide ideation risk. Although this preliminary finding is first in need of further empirical study and replication, implications may be indicated for both prevention and intervention efforts. In particular, this finding may indicate that children identified as experiencing emotional abuse are an important target population for early suicide prevention efforts. Specifically, prevention efforts aimed at addressing early emotion regulation skills and adaptive/effective strategy use may be of critical to mitigating the development of limited ERS in adolescence. Dually, current findings point to the importance of early evidence-based, trauma-focused intervention for youth identified as experiencing emotional abuse, and their caregivers. Evidence-based treatments, such as TF-CBT (47) and Child-Parent Psychotherapy [CPP; Ghosh (70)] that flexibly and sensitively address trauma psychoeducation, affect regulation, parenting practices, and increase safety and security in the child-caregiver attachment relationship are clinically indicated.

## Data Availability Statement

The data analyzed in this study is subject to the following licenses/restrictions: Data was collected as part of a larger IRB-approved study and data is maintained in accordance with the IRB-approved protocol. Requests to access these datasets should be directed to csharp2@central.uh.edu.

## Ethics Statement

The studies involving human participants were reviewed and approved by Institutional Review Boards at University of Houston and Baylor College of Medicine. Written informed consent to participate in this study was provided by the participants' legal guardian/next of kin.

## Author Contributions

CH and ES conceived the presented empirical research questions, focus of the current manuscript, and wrote the manuscript with support from CS. CS conceived and principally investigated the overarching study, including supervising the study and its implementation. CH and ES conducted the analyses. All authors provided critical input to the manuscript writing and final manuscript.

## Conflict of Interest

The authors declare that the research was conducted in the absence of any commercial or financial relationships that could be construed as a potential conflict of interest.
